# Report of Prof. Valentine Mott’s Surgical Clinics in the University of New York

**Published:** 1860-09

**Authors:** 


ART. XI.—*Report of Professor Valentine Mott's Surgical Clinics in the University of New York; Session* 1859–60. By SAMUEL W. FRANCIS, Member of Dr. Mott's Surgical Staff. 12mo., pp. 209. New York: S. S. Wood, 1860.

THE little volume before us will be sought after with avidity by the many friends and pupils of the illustrious surgical chieftain of New York, whose zeal in his profession, although he has passed the age of threescore years and ten, is as keen and vigorous as it was at the commencement of his brilliant career. As the title denotes, it consists of a report of the principal cases treated by Professor Mott at the surgical Clinic of the University of New York during the session of 1859–60, and is well worthy of an attentive perusal, inasmuch as it embodies the practical views of a man who has long enjoyed a world-wide reputation as one of the greatest surgeons of the age. The young editor has performed his task with commendable zeal and ability, thus affording an earnest of future usefulness and fame, destined, we trust, to rival those of his illustrious father, who, to indomitable industry and perseverance, and an ardent love for his profession, adds the character of the medical philosopher and the varied accomplishments of the scholar.

We subjoin the following extract, the only one which our limited space will admit of, as a specimen of the style, and the nature of the matter in this Report. The case to which it refers is a type of what so often happens in the hands of inexperienced and careless practitioners:—

"A young boy, aged four years, was then brought into the Clinic and shown to the students, who had been operated upon for stone in the bladder, some three weeks before, by Dr. Alexander B. Mott. The child had suffered much from pain in the hypogastric region, had experienced great difficulty at times in passing his urine, and occasionally, while micturating, it would stop on a sudden, as if some impediment of a mechanical nature had arrested the flow; having, as it were, fallen in front of the urethra. At times, moreover, there had been a kind of sediment deposited in the water which he had passed; and it was a constant source of anxiety to his parents that his urine should have so much thick and viscid material in it. This was no doubt the result of inflammation, occasioned by the irritation of the internal coat of the bladder, brought about by the attrition of a foreign body, which resulted in an increase of mucous secretion. This deposit in the urine, after excessive drinking of stimulating liquors, has not unfrequently caused the student to suspect the presence of albumen. But the nitric acid test, or the application of heat to a small portion in a test tube, or an additional trial of a solution of the bichloride of mercury, would at once reveal the truth of the case, and calm all apprehension on the part of the medical man.

"Several practitioners had made careful examinations with the sound, but could ascertain nothing satisfactory or conclusive in the case before them. There was much that was rendered obscure in the present instance. The rational signs were indicative of the presence of stone. While that which was all-important to confirm the opinion of a few, namely, the sound, failed in elucidating the matter or unraveling the mystery. The case was abandoned, and Dr. A. B. Mott was called in to treat the patient for the symptoms as exhibited in the continued sufferings of the young boy. He sounded very carefully, and succeeded in turning the instrument behind the prostatic portion of the urethra. Moreover, by passing the sound far up into the bladder,—bearing in mind that in a child there is no 'bas fond,' while in a person advanced in years it increases, and, in fact, in the old man there is quite a large sac below the sphincter of the neck of the bladder; again, in one of few years the bladder is more perpendicular and farther to reach,—the doctor ascertained, by following out these principles, the presence of a very small calculus, which he could distinctly hear click when the steel came in contact with its hard exterior."

